# Metrics of Antifungal Effects of Ciprofloxacin on *Aspergillus fumigatus* Planktonic Growth and Biofilm Metabolism; Effects of Iron and Siderophores

**DOI:** 10.3390/jof8030240

**Published:** 2022-02-28

**Authors:** Gabriele Sass, Lynn Scherpe, Marife Martinez, Julianne J. Marsh, David A. Stevens

**Affiliations:** 1 California Institute for Medical Research, San Jose, CA 95128, USA; lynnscherpe@gmail.com (L.S.); mmartinez@cimr.org (M.M.); juliannejmarsh@gmail.com (J.J.M.); stevens@stanford.edu (D.A.S.); 2 Faculty of Science and Engineering, Maastricht University, 6229 EN Maastricht, The Netherlands; 3 Division of Infectious Diseases and Geographic Medicine, Department of Medicine, Stanford University School of Medicine, Stanford, CA 94305, USA

**Keywords:** *Aspergillus*, *Pseudomonas*, co-infection, antifungal therapy, ciprofloxacin, cystic fibrosis, iron, biofilm

## Abstract

*Pseudomonas aeruginosa* and *Aspergillus fumigatus* frequently coexist in the airways of immunocompromised patients or individuals with cystic fibrosis. Ciprofloxacin (CIP) is a synthetic quinolone antibiotic commonly used to treat bacterial infections, such as those produced by *Pseudomonas aeruginosa.* CIP binds iron, and it is unclear what effect this complex would have on the mycobiome. The effects of CIP on *Aspergillus* were dependent on the iron levels present, and on the presence of *Aspergillus* siderophores. We found that CIP alone stimulated wildtype planktonic growth, but not biofilm metabolism. At high concentrations, CIP antagonized a profungal effect of iron on wildtype *Aspergillus* metabolism, presumably owing to iron chelation. CIP interfered with the metabolism and growth of an *Aspergillus* siderophore mutant, with the effect on metabolism being antagonized by iron. CIP acted synergistically with iron on the growth of the mutant, and, to a lesser extent, the wildtype. In summary, CIP can increase fungal growth or affect fungal metabolism, depending on the local iron concentration and available siderophores. Therefore, high local CIP concentrations during treatment of *Pseudomonas–Aspergillus* co-infections may increase the fungal burden.

## 1. Introduction

*A. fumigatus* and *P. aeruginosa* share the same polymicrobial niches, e.g., in the lungs of immunocompromised patients and people with cystic fibrosis (CF), aggravating infections [1,2,3,4]. Interactions of *A. fumigatus* and *P. aeruginosa* have been the topic of extensive research [5,6,7,8,9,10]. However, limited studies have focused on how drugs prescribed for one of these pathogens can affect the other pathogens present in the same microenvironment.

Both *A. fumigatus* and *P. aeruginosa* require and compete for iron for their growth, metabolism, and virulence, and produce siderophores for its uptake [10,11,12]. In people with CF, different areas of the lungs have different concentrations of iron; in mucoid plugs, for instance, iron levels are low, while in other areas, e.g., caused by hemorrhage, an increased iron content is present [13,14]. The iron content in the microenvironment plays an important role in *A. fumigatus* and *P. aeruginosa* interactions, but also in their response to antibiotics and antifungals [14]. 

Quinolones are broad-spectrum antibiotics that act against both Gram-positive and Gram-negative bacteria [15]. Most studies previously conducted on ciprofloxacin (CIP) focused solely on its ability to eradicate *P. aeruginosa*, and ignored the potential effects it may have on other pathogens, as well as the effect of essential minerals, such as iron, coexisting in the niche [16,17]. The effects of CIP, especially in combination with iron, on the growth and metabolism of *A. fumigatus* have not been studied in detail.

In addition, it has been shown that *Pseudomonas* is able to produce a variety of quinolone compounds that have antifungal activities, such as the *Pseudomonas* quinolone signal [PQS] [8,10]. These molecules act as quorum-sensing signal molecules; they control the expression of virulence factors, upregulate *Pseudomonas* genes, are responsible for siderophore production, and, most relevantly, they act as iron chelators [18]. Their high affinity for iron allows them to bind and transport it to the bacterial cell membrane, thereby reducing the availability of free iron in the environment. Therefore, under iron-limiting conditions, PQS inhibits the metabolism of forming and preformed *A. fumigatus* biofilms, as well as planktonic growth [8,10]. Under non-limiting iron conditions, however, the growth and biofilm formation of *A. fumigatus* was enhanced, a feature dependent on the presence of *Aspergillus* siderophores, suggesting that interactions between PQS and siderophores enhance iron uptake by the fungus [8,10].

Here, we investigated the effects of CIP on the growth and metabolism of *A. fumigatus*. CIP is a fluoroquinolone antibiotic prescribed to treat a large array of bacterial infections, including those produced by *P. aeruginosa* [19].

## 2. Materials and Methods

Materials: Ciprofloxacin (CIP, molecular mass: 331.346 g/mol), ferric iron (FeCl_3_), 2,3-bis[2-methoxy-4-nitro-5-sulfophenyl]-2H-tetrazolium-5-carboxanilide inner salt (XTT), menadione, and RPMI 1640 medium were purchased from Sigma-Aldrich (St. Louis, MO, USA). The iron content in RPMI 1640 medium was below the detection limit (<1 µM, measured by inductively coupled plasma optical emission spectroscopy by Paolo Visca, Rome, Italy, personal communication). 

Strains and isolates: The fungal strains used in this study are shown in Table 1. The use of all microbes in our laboratory is approved by the CIMR Biological Use Committee (approval no. 001-03Yr.16).

*Aspergillus* forming biofilm metabolism assay: Test substances were combined in equal parts by volume (25 µL each) in sterile flat-bottom 96-well culture plates to prepare the final concentrations indicated in the figures and figure legends. RPMI 1640 medium served as the negative control. *A. fumigatus* conidia (10^5^/mL final concentration for the wildtype, or 2 × 10^5^/mL final concentration for the siderophore-deficient mutant) were distributed into each well at 50 µL/well. The mutant was used at a higher conidia concentration to assure hyphal growth similar to the wildtype, as measured in RPMI controls, without the addition of drugs. The equal growth of controls at the end of the comparative experiments was verified microscopically, as well as by XTT measurements. As an example, the XTT measurements at 490 nm for the control (no iron, no CIP) wells on three 96-well plates per fungus were as follows: AF13073 wildtype: 0.203, 0.215, and 0.241; SidA mutant: 0.192, 0.252, and 0.243. Each plate was normalized to its individual control, and the mean for all 3 plates per fungus was regarded as the result for one experiment. Final volumes in the wells during the assays were 100 µL. The assay plates were incubated at 37 °C overnight. All biofilm assays were evaluated by an XTT metabolic assay, as detailed previously [5,23]. Briefly, 150 µL of an XTT/menadione mixture (150 µg/mL XTT, 30 µM menadione) was added to each test well and incubated at 37 °C for about one hour. Supernatants from each well were transferred to a fresh 96-well plate (100 µL) and assayed using a plate reader (Vmax, Molecular Devices, San Jose, CA, USA) at 490 nm. All results were calculated as percent of the RPMI control present in each individual experiment, set at 100%. We also performed a preliminary experiment, where we incubated CIP with the XTT reaction liquid to try to determine whether CIP affects the XTT reaction. We did not observe differences in the measurements at 490 nm between the control wells, containing only the XTT reaction liquid, and the wells containing different concentrations of CIP.

Assay for visual measurement of *Aspergillus* planktonic growth: The test substances were combined in equal parts by volume (50 µL each), in sterile 4 mL culture tubes, to prepare the final concentrations indicated in the figures and figure legends. The RPMI 1640 medium served as the negative control. A total of 900 µL of *A. fumigatus* inoculum, prepared according to the CLSI guidelines for *Aspergillus* broth macro-dilution, was added to the tubes. The tubes were incubated for 48 h at 35 °C. The amount of *A. fumigatus* planktonic growth observed in each tube was examined visually, and reviewed by 3–5 of the authors. Following the CLSI protocol, a 4+ reading was assigned to the control tube (RPMI with *A. fumigatus*, no test substance) at the time of reading. At this time, all other tubes were compared to the control. Readings from 0 to 3+ were assigned to all tubes that appeared to have less *A. fumigatus* growth than the control, while a reading of 0 indicated no visible *A. fumigatus* growth. For research purposes, 5+ or 6+ readings were assigned to all tubes containing increasing levels of *A. fumigatus* growth compared to the control.

In many experiments, we compared spectrophotometric readings of planktonic growth with visual readings. We found that spectrophotometric readings of growth were more difficult to analyze and were less sensitive than visual readings for the filamentous fungus. The fungus tends to stick to the sides of the tubes, causing poor spectrophotometric reproducibility. Although spectrophotometric readings grossly concurred with visual readings, for the reasons stated, we utilized visual assessments of fungal growth.

Optical evaluation of fungal growth on filter paper: Following visual measurement of *A. fumigatus* planktonic growth in 1 mL of volume, 1 mL of XTT solution (150 µg/mL XTT, 30 µM menadione) was added to each tube, and the tubes were incubated at 35 °C for approximately one hour. The stained contents of the tubes were transferred onto filter paper. The filter papers were air-dried, and a photograph was taken. 

Reagent interaction studies: Two-dimensional reagent interaction (“checkerboard”) studies were performed, as previously detailed [24], and the concentrations studied are delineated in the Results Section 3.

Statistical analysis: Each assay was performed several times, as indicated in the figure legends. The results were analyzed by Student’s *t*-test when two groups were compared, and by 1-way ANOVA, combined with a Tukey’s post-test, for multiple comparisons. All statistical data in this study are expressed as mean ± SD.

## 3. Results

### 3.1. CIP Reduces A. fumigatus Biofilm Metabolism in the Absence of Siderophores

**Figure 1:**
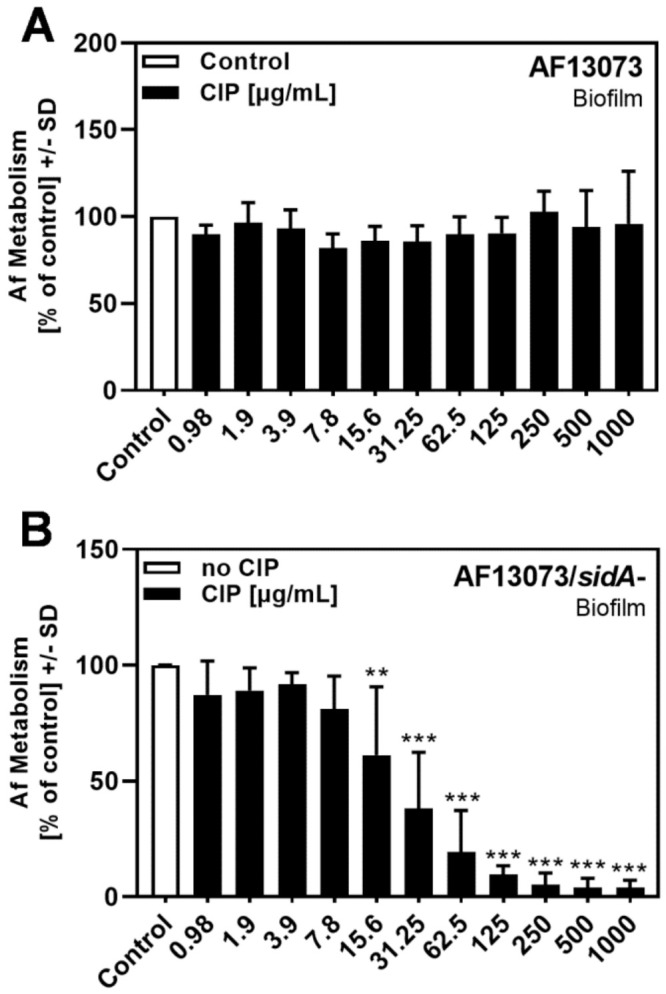
CIP reduces *A. fumigatus* biofilm metabolism in the absence of siderophores. CIP (final concentrations 0.98–1000 µg/mL in RPMI 1640 medium, x-axis) was combined with (**A**) AF13073 conidia (10^5^ conidia/mL in RPMI 1640 medium) or (**B**) AF13073/*sidA*- conidia (2 × 10^5^ conidia/mL in RPMI 1640 medium). As the siderophore-deficient mutant has slower growth, using double the amount of conidia assured similar growth to the wildtype in the untreated controls for both fungi. Assay plates were incubated at 37 °C overnight. Fungal metabolism was measured by an XTT assay. The metabolism in the presence of RPMI alone was regarded as 100%, and was compared to each supernatant dilution. Statistical analysis, 1-way ANOVA: two or three asterisks = *p* ≤ 0.01 or *p* ≤ 0.001, respectively. Comparison: RPMI (white bar) vs. all other bars (black bars). A: *n* = 6–7; B: *n* = 4–5.

The natural fluoroquinolone PQS has been shown to interfere with the metabolism and growth of *A. fumigatus* under iron-limiting conditions [8,10]. Using the synthetic fluoroquinolone CIP under the same iron-limiting conditions, created by the use of the RPMI 1640 medium, in which iron is not detectable (<1 µM), CIP did not affect the metabolism of the *A. fumigatus* wildtype (AF13073) biofilm at concentrations up to 1 mg/mL (Figure 1A). In contrast, the metabolism of the siderophore-deficient mutant of AF13073 (AF13073/*sidA*-) was reduced significantly at CIP concentrations of 15.6 µg/mL or higher (up to 1 mg/mL) (Figure 1B). 

### 3.2. CIP Induces A. fumigatus Planktonic Wildtype Growth, but Reduces Planktonic Growth of a Siderophore-Deficient Mutant

**Figure 2:**
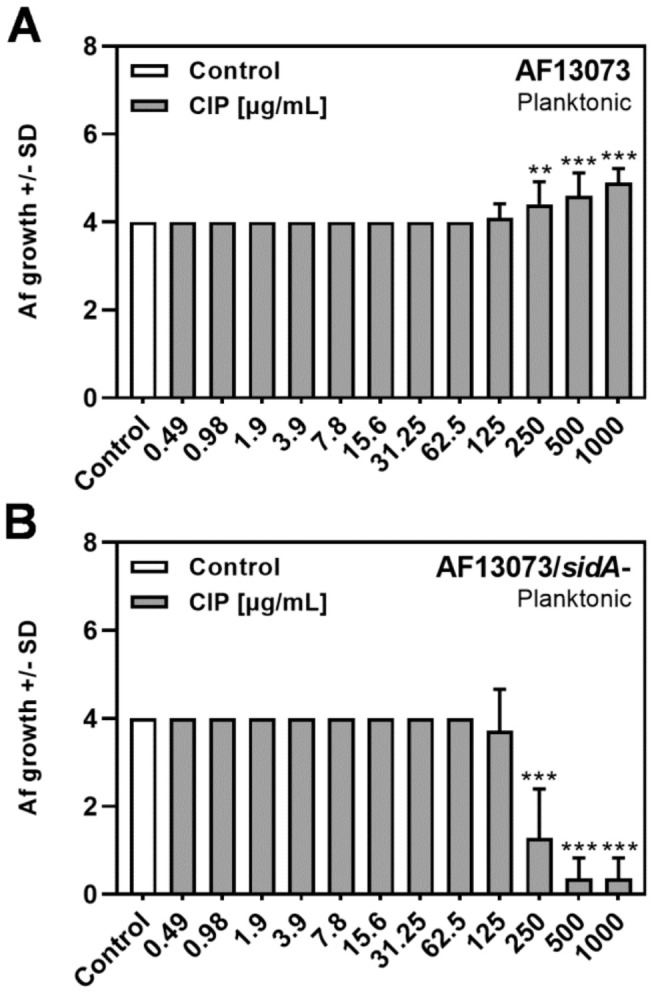
CIP induces *A. fumigatus* planktonic wildtype growth, but reduces planktonic growth of the siderophore-deficient mutant. CIP (final concentrations 0.49–1000 µg/mL in RPMI 1640 medium, x-axis) was combined with (**A**) AF13073 or (**B**) AF13073/*sidA*- conidia according to the CSLI guidelines for macro-dilution. The tubes were incubated at 37 °C for 48 h. Fungal growth was measured visually, and rated as described in the Material and Methods section, with growth in control tubes assigned a ‘4’. The tubes with less growth were rated a number between 0 and 3, and for tubes with more growth than the control, a number 5 or 6, depending on relative amount of growth. Statistical analysis, 1-way ANOVA: two or three asterisks = *p* ≤ 0.01 or *p* ≤ 0.001, respectively. Comparison: RPMI (white bar) vs. all other bars (grey bars). A: *n* = 8–10; B: *n* = 7.

In contrast to the findings for biofilm metabolism (Figure 1), high concentrations of CIP (250 µg/mL up to 1 mg/mL) showed profungal effects on AF13073 wildtype planktonic growth (Figure 2A). The same concentrations had antifungal effects on the planktonic growth of the siderophore-deficient fungus (Figure 2B).

**Figure 3:**
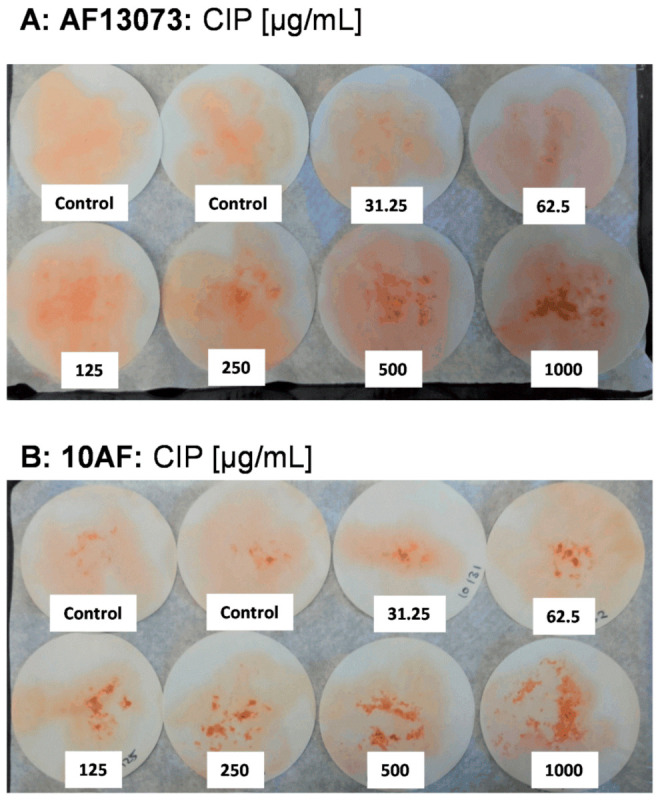
CIP induces planktonic growth of A. fumigatus wildtype strains AF13073 (**A**) and 10AF (**B**). After 48 h of planktonic growth in the presence of CIP (final concentrations 31.25–1000 µg/mL in RPMI 1640 medium), tubes were diluted 1:2 with XTT solution, incubated at 37 °C for 30 min, and the complete content of each tube was spread on filter paper.

The profungal effects of CIP on planktonic growth were also visualized after 48 h of growth, by staining and plating the wildtype fungus on filter paper. The AF13073 wildtype showed visually enhanced growth ≥250 µg/mL (Figure 3A), and the 10AF wildtype showed visually enhanced growth ≥62.5 µg/mL (Figure 3B). Compared to the control = 100% (RPMI, no drug), CIP (62.5 ug/mL) increased the biomass by 26% (AF13073) and 78% (10AF). No further increase in the biomass was observed when ≥250 ug/mL CIP was used (maximum increase in the biomass over control was as follows: AF13073:186%, 10AF: 105%). 

### 3.3. Iron Induces the Metabolism and Growth of A. fumigatus

**Figure 4:**
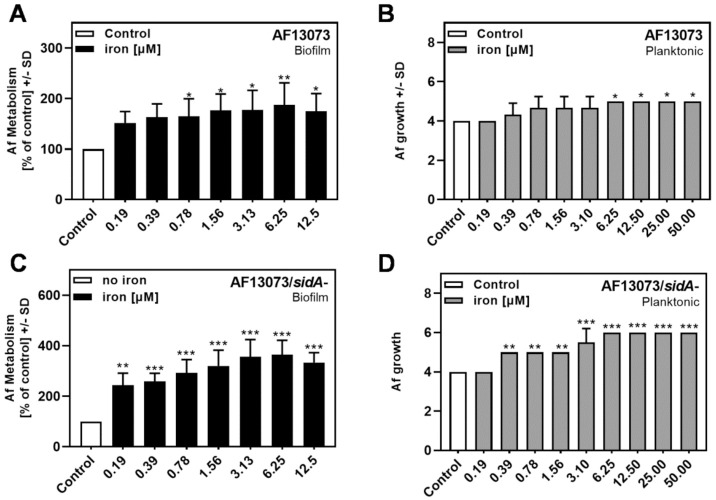
Iron induces the metabolism and growth of *A. fumigatus*. FeCl_3_ (iron; final concentrations 0.19–12.5 µM in RPMI 1640 medium, x-axis) was combined with (**A**,**B**) AF13073 conidia or (**C**,**D**) AF13073/*sidA*- conidia. (**A**,**C**) For the measurement of biofilm metabolism, plates were incubated at 37 °C overnight. Fungal metabolism was measured by an XTT assay. (**B**,**D**) The tubes for planktonic fungal growth were incubated at 37 °C for 48 h. Fungal growth was measured visually, and rated as described in the Material and Methods section. Biofilm metabolism or planktonic growth in the presence of RPMI alone was compared to each iron concentration. Statistical analysis, 1-way ANOVA: one, two or three asterisks = *p* ≤ 0.05, *p* ≤ 0.01 or *p* ≤ 0.001, respectively.

Iron is a crucial factor for the growth and metabolism of *Aspergillus* [9,10,12]. Our results confirmed this finding, showing significantly increased AF13073 wildtype metabolism (Figure 4A) and growth (Figure 4B) ≥0.78 and 6.25 µM, respectively. The siderophore-deficient *Aspergillus* mutant was more responsive to iron than its wildtype, showing significantly increased metabolism ≥0.19 µM (Figure 4C), which was the lowest concentration tested, and significantly increased growth from 0.39 µM (Figure 4D). 

### 3.4. Iron and CIP Interact and Affect A. fumigatus Biofilm Metabolism

**Figure 5:**
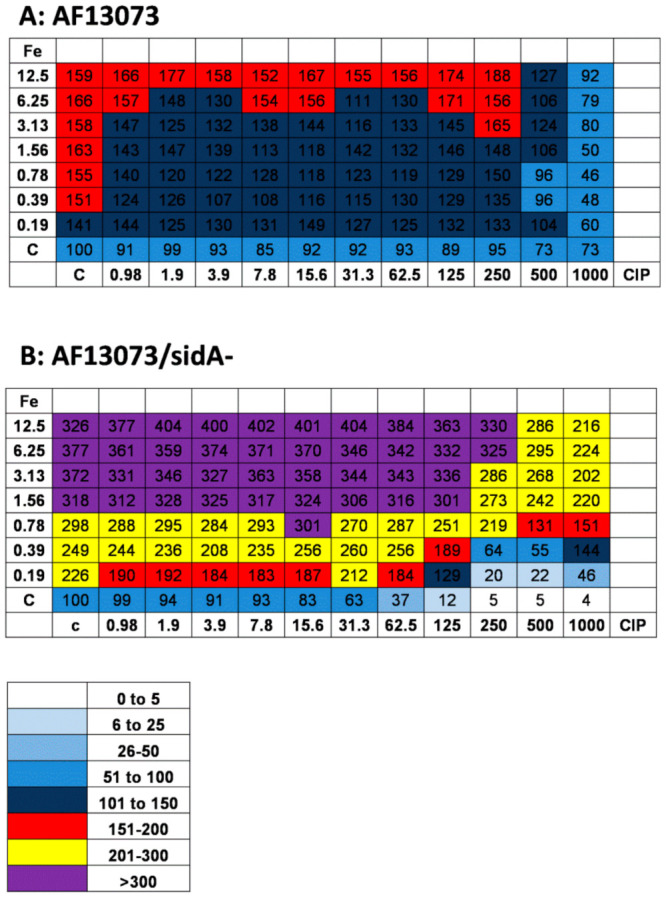
Iron and CIP interact and affect *A. fumigatus* biofilm metabolism. CIP (final concentration 0.98–1000 µg/mL in RPMI 1640 medium) was combined with FeCl_3_ (iron; final concentration 0.19–12.5 µM in RPMI 1640 medium) using (**A**) AF13073 conidia (10^5^ conidia/mL in RPMI 1640 medium) or (**B**) AF13073/*sidA*- conidia (2 × 10^5^ conidia/mL in RPMI 1640 medium). As the siderophore-deficient mutant has slower growth, using double the amount of conidia assured similar growth to the wildtype in the untreated controls for both fungi. The assay plates were incubated at 37 °C overnight. Fungal metabolism was measured by an XTT assay. Metabolism in the presence of RPMI alone was regarded as 100%, and compared to each supernatant dilution. The color chart visualizes groups of iron/CIP combinations with similar growth. A: *n* = 2; B: *n* = 2.

When CIP and iron were combined, CIP concentrations as low as 0.98 µg/mL interfered with the profungal effects of iron on the AF13073 wildtype, from 0.19 µM iron to about 3 µM (Figure 5A). At high concentrations of CIP, 500 and 1000 µg/mL, the profungal effects of iron were almost abolished (Figure 5A). On the siderophore-deficient fungus, the iron antagonized the antifungal effects of CIP in a dose-dependent manner (Figure 5B). High concentrations of CIP (500 and 1000 µg/mL) reduced the profungal effects of iron, even if the iron was used at the highest concentration tested here, 12.5 µM (Figure 5B).

### 3.5. Iron Does Not Further Increase Profungal Effects of CIP on A. fumigatus Planktonic Growth

**Figure 6:**
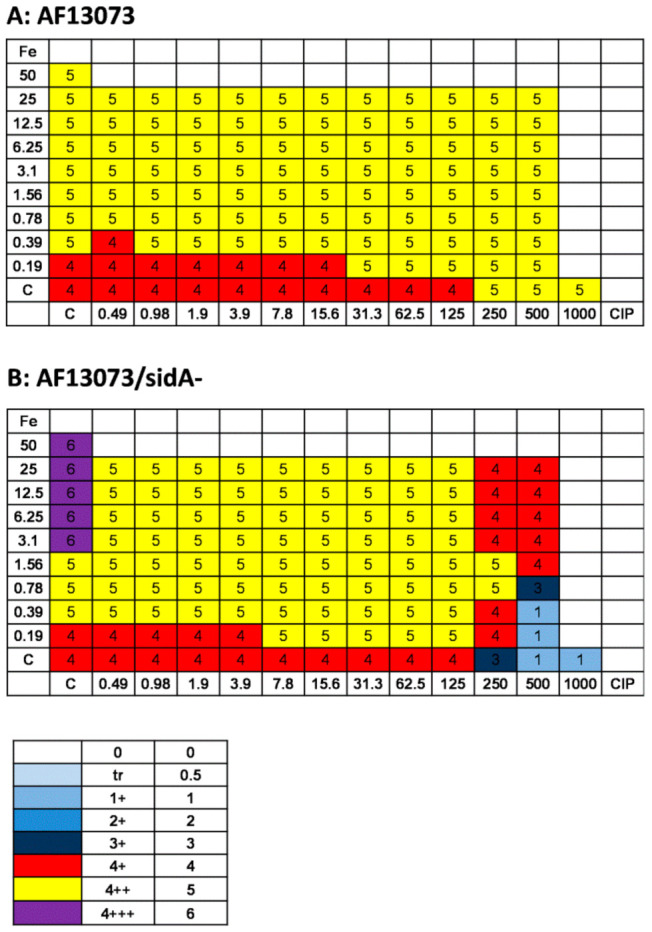
Iron does not further increase the profungal effects of CIP on the planktonic growth of *A. fumigatus*. CIP (final concentrations 0.49–1000 µg/mL in RPMI 1640 medium) was combined with FeCl_3_ (iron; final concentration 0.19–12.5 µM in RPMI 1640 medium) using (**A**) AF13073 or (**B**) AF13073/*sidA*- conidia, according to CSLI guidelines for macro-dilution. The tubes were incubated at 37 °C for 48 h. Fungal growth was measured visually, and rated as described in the Material and Methods section, with the growth in control tubes being assigned a ‘4’, as per CLSI methodology. The tubes with less growth were rated a number between 0 and 3, and for the tubes with more growth than the control, a number 5 or 6, proportional to the amounts of growth. The color chart visualizes groups of iron/CIP combinations with the same growth. A: *n* = 2; B: *n* = 2.

High concentrations of CIP alone (Figure 2A) or iron alone ≥6.25 µM (Figure 4B) had profungal effects on the AF13073 wildtype. When CIP and iron were combined, there was no further increase in growth, except for the combination of 0.19 µM iron with CIP at 31.3–125 µg/mL (Figure 6A). On the siderophore-deficient *Aspergillus*, the combination of iron at 0.19 µM with CIP at 7.8 to 125 µg/mL resulted in increased growth, compared to each factor alone (Figure 6B). CIP interfered with the profungal effects of the higher iron concentrations (Figure 6B). The antifungal effects of CIP (250 µg/mL or higher) were antagonized by iron (Figure 6B). 

## 4. Discussion

We confirmed our prior studies findings, which showed that iron stimulates the metabolism and growth of the wildtype [8,25]. The profungal effects of CIP on the wildtype fungus were only detected at concentrations ˃125 µg/mL, and for planktonic growth, but not for biofilm metabolism. These profungal effects on planktonic growth were observed by two different methods, and on two wildtype strains, AF13073 and 10AF. It would enhance our findings if more *Aspergillus* strains were studied. An explanation for the discrepancy between the CIP effects on planktonic growth and biofilm metabolism might be that fungal biofilms are more drug resistant, making this feature one of the pathogen’s most important virulence factors, interfering with eradication of the infection [26,27]. In addition, the XTT assay assessed the mitochondrial oxidative metabolism. CIP appears to have an effect on the enzymes affecting the growth of the planktonic wildtype *A. fumigatus*, but may have a differential effect on the dehydrogenase enzymes that are involved in mitochondrial respiration [28,29]. Furthermore, it is speculated that CIP dysregulates electron transfer, presenting an additional reason for the discrepancy between the XTT and growth results [30].

CIP antagonizes the profungal effect of iron on wildtype metabolism, presumably by CIP chelation of iron. CIP has a far lesser effect on the stimulation of wildtype planktonic growth by iron; some synergy was observed, suggesting that there may be some CIP assistance in delivering iron to *A. fumigatus.*

In the case of the *A. fumigatus* that lacks siderophores, CIP has a profound depressing effect on both the metabolism and planktonic growth, presumably by severe iron denial. These putative mechanisms of CIP action require further study.

The effects of CIP on biofilm metabolism are stronger than the effects on planktonic growth. It has been shown that *Aspergillus* biofilms are very sensitive to iron starvation [7,10]. Supplying external iron alone has a much greater stimulatory effect on the siderophore mutant than on the wildtype, likely because the mutant can now take advantage of other possible means of acquiring iron from its environment, including diffusion and oxidation–reduction to Fe^2+^. Iron antagonizes the antifungal effects of CIP on metabolism, and this suggests that the added iron can supply some of the iron that CIP would otherwise deny. However, at high CIP concentrations, the stimulatory effect of iron on metabolism is antagonized (as was observed with the wildtype), and we hypothesize that these high concentrations can produce an iron deficit by excessive iron chelation.

The discovery of naturally occurring quinolones led to the development of synthetic quinolone drugs, such as CIP [18]. CIP, a fluoroquinolone, is a bactericide that inhibits the enzymes DNA topoisomerase and DNA gyrase, involved in bacterial DNA replication [31]. Fungi possess high levels of topoisomerase I and II [32], making them possible targets for CIP. 

Quinolone drugs, other than CIP, were previously examined for their effects on *A. fumigatus* growth. These studies showed that quinolones can inhibit fungal growth by interacting with topoisomerase II [32,33]. One study also found that the quinoline bromoquinol exhibits broad-spectrum antifungal activity by inducing oxidative stress and apoptosis in *A. fumigatus* [34]. Another study that examined the effects of CIP on numerous fungi, not including *A. fumigatus*, found no inhibitory effects on *H. capsulatum* and *C. posadasii* growth [33], and Stergiopoulou et al. [32] discovered that CIP was even able to increase *A. fumigatus* growth, but they did not postulate a mechanism of action. 

The CIP concentrations >125 µg/mL, triggering increased planktonic fungal growth, were well above the therapeutic serum range of up to 7.27 mg/L, which can be achieved by taking CIP orally; this is the most common form of administration [35]. However, recent studies have shown that via other methods of administration, higher CIP concentrations can be deposited at the site of infection. A clinical study performed on pneumonia patients, where the infection occurs in the alveolar spaces and pulmonary interstitium, found that CIP concentrations could reach up to 34.9 µg/mL in alveolar macrophages, measured four hours after intravenous infusion of 400 mg CIP [36]. Inhaling CIP deposits even higher concentrations in the airways of CF patients [37,38]. Therefore, these forms of CIP administration pose greater risks of promoting *A. fumigatus* during co-infections.

It is known that oral iron reduces quinolone bioavailability [39,40], as a result of iron binding to ciprofloxacin and complexation. Kara et al. [41] found that ciprofloxacin increases the rate of ferrous iron [Fe^2+^] oxidation, and thereupon binds rapidly to the resulting ferric iron [Fe^3+^] to form a CIP–Fe complex. These complexes are poorly absorbed by the body, and decrease the amount of CIP and bioavailable iron. In consequence of the reduced iron availability, one might assume that antifungal effects are taking place. However, in our study, the combination of CIP and iron increased the growth of the wildtype *A. fumigatus* modestly more than each reagent alone. In future studies, it would be interesting to investigate the effects of CIP on *Aspergillus* siderophore production. Iron complexation might increase siderophore production, which would allow more iron uptake by the fungus, and more growth. 

Iron levels in certain areas of CF patient lungs are already elevated [42], supporting fungal growth, but the present study determined that iron concentrations as low as 0.2 µM, in combination with 15.6 µg/mL CIP, are sufficient to enhance the growth of the wildtype *A. fumigatus*. These results may suggest that CIP can act similarly to PQS, by feeding iron to *A. fumigatus*, via interactions with *A. fumigatus* siderophores. In previous studies, we found that PQS can boost the growth of *A. fumigatus* in high-iron environments, and that, for this to happen, *A. fumigatus* siderophores must be present [8]. The prolonged residence of microbes in chronic infections, a changing iron milieu, and the competition or cooperation with other members of the microbiome, can induce mutations that downplay the need for energy-consuming siderophore production [2]. 

## 5. Impact

Previous studies have focused on the effects of naturally occurring quinolones on *A. fumigatus*; however, such studies have paid less attention to the possible effects of clinically prescribed synthetic quinolones on this pathogen. As bacteria and fungi commonly coexist in the airways of immunocompromised patients and people with CF, it is important that the effect of each clinically prescribed antibiotic or antifungal is tested on all microorganisms that may be present in the target niche. The present study revealed that the antibiotic ciprofloxacin can potentially stimulate *A. fumigatus* growth in the airways of immunocompromised patients and people with CF, especially in areas where iron levels are high. Therefore, while eradicating the bacterial infection, CIP treatment could worsen a coexisting *Aspergillus* infection, and, thereby, the overall health of the patient. This research should make clinicians aware of the risks associated with prescribing CIP.

## Figures and Tables

**Table 1:** *A. fumigatus* strains and isolates used in this study.

Organism	Isolate	Description	ATCC	Ref.
*A. fumigatus*	AF13073	Parental strain of AF13073/*sidA*-	13073	
*A. fumigatus*	AF13073/*sidA*-	l-ornithine-*N* 5-mono-oxygenase deficient *Af* mutant strain		[20]
*A. fumigatus*	10AF	Virulent patient isolate	90240	[21,22]

## Data Availability

Data are available from the corresponding author upon request.
